# Immune, metabolic landscapes of prognostic signatures for lung adenocarcinoma based on a novel deep learning framework

**DOI:** 10.1038/s41598-023-51108-x

**Published:** 2024-01-04

**Authors:** Shimei Qin, Shibin Sun, Yahui Wang, Chao Li, Lei Fu, Ming Wu, Jinxing Yan, Wan Li, Junjie Lv, Lina Chen

**Affiliations:** College of Bioinformatics Science and Technology, Harbin Medical University, Harbin, 150000 China

**Keywords:** Cancer metabolism, Cancer microenvironment, Cancer models, Cancer therapy, Lung cancer, Data mining, Machine learning

## Abstract

Lung adenocarcinoma (LUAD) is a malignant tumor with high lethality, and the aim of this study was to identify promising biomarkers for LUAD. Using the TCGA-LUAD dataset as a discovery cohort, a novel joint framework VAEjMLP based on variational autoencoder (VAE) and multilayer perceptron (MLP) was proposed. And the Shapley Additive Explanations (SHAP) method was introduced to evaluate the contribution of feature genes to the classification decision, which helped us to develop a biologically meaningful biomarker potential scoring algorithm. Nineteen potential biomarkers for LUAD were identified, which were involved in the regulation of immune and metabolic functions in LUAD. A prognostic risk model for LUAD was constructed by the biomarkers HLA-DRB1, SCGB1A1, and HLA-DRB5 screened by Cox regression analysis, dividing the patients into high-risk and low-risk groups. The prognostic risk model was validated with external datasets. The low-risk group was characterized by enrichment of immune pathways and higher immune infiltration compared to the high-risk group. While, the high-risk group was accompanied by an increase in metabolic pathway activity. There were significant differences between the high- and low-risk groups in metabolic reprogramming of aerobic glycolysis, amino acids, and lipids, as well as in angiogenic activity, epithelial-mesenchymal transition, tumorigenic cytokines, and inflammatory response. Furthermore, high-risk patients were more sensitive to Afatinib, Gefitinib, and Gemcitabine as predicted by the pRRophetic algorithm. This study provides prognostic signatures capable of revealing the immune and metabolic landscapes for LUAD, and may shed light on the identification of other cancer biomarkers.

## Introduction

Cancer is still a global major public health problem^1^. According to the latest global cancer statistics estimates, lung cancer remains the leading cause of cancer death and the second most commonly diagnosed cancer, accounting for approximately 20% of cancer-related mortality and 10% of incidence^2^. Non-small cell lung cancer (NSCLC) accounts for approximately 85% of lung cancer cases^3^, with lung adenocarcinoma (LUAD) as its main histologic subtype and its incidence is still increasing^4^. Despite significant advances in diagnosis and treatment, the 5-year survival rate of LUAD is only 4–17%^5^. Therefore, screening and poor prognosis of lung adenocarcinoma remains an ongoing challenge. Accumulating evidence suggests that biomarker identification and application are of major importance for timely diagnosis and accurate prognosis of cancer^6,7^.

Machine learning has been widely used in biomarker discovery studies for cancer^8–10^. For example, based on breast cancer gene expression datasets, Zare et al.^11^ identified 59 novel inflammatory breast cancer-specific gene signatures using a random forest approach. Zhang et al.^12^ used a support vector machine algorithm to excavate a m6A target miRNAs diagnostic signature for cancer detection and successfully implemented it for lung cancer. Machine learning algorithms were deployed to develop tumor-infiltrating immune cell associated RNAs to predict survival outcomes in LUAD patients^13^. Deep learning has gained increasing attention in the research of cancer signatures identification^14,15^. The denoising autoencoder, an unsupervised deep learning algorithm, has been successfully applied to LUAD molecular signature mining^16^. Divate et al.^17^ presented a robust neural network model providing gene signatures for accurate classification of cancers based on gene expression data. Three feed-forward neural networks were employed to RNA-seq samples from 18 solid tumor types and recognized transcriptome signatures that were consistent across tumors^18^. Deep learning methods are capable of identifying highly complex patterns in large datasets compared to common machine learning techniques, which help to efficiently identify molecular signatures associated with cancer^19^.

Variational autoencoder (VAE) is a deep generative model based on variational Bayesian inference designed to learn nonlinear latent representations of high-dimensional data^20^. VAE has shown encouraging results in capturing biologically meaningful low-dimensional representations of multi-omics data. Daniel et al.^21^ utilized VAE to learn a generalized latent representation of large-scale metabolomics data, and VAE representation outperformed both linear and nonlinear principal component analysis. VAE models trained on gene expression data have good ability to identify generalizable biological representations^22^. In addition, VAE has been applied in multi-omics representation learning such as proteomics and epigenomics^23,24^. Evidently, VAE is a powerful method for dimensionality reduction. Neural networks are commonly employed in the current deep learning field, among which multilayer perceptron (MLP) is widely used in cancer diagnosis research. Lorencin et al.^25^ combined MLP and Laplacian edge detector to achieve bladder cancer detection. Deep learning methods including MLP have revealed salivary glycopatterns as biomarkers for the diagnosis of papillary thyroid cancer^26^. MLP has also been applied to screening for breast, colorectal, and prostate cancers^27–29^. Shapley Additive Explanations (SHAP) is derived from the Shapley value in cooperative game theory, which aims to tackle the problem of lack of interpretability faced by machine learning^30^. In a study by Chakraborty et al.^31^, SHAP facilitated the identification of prognostic factors for breast cancer by enhancing the interpretability of the extreme gradient boosting model. Multiple studies have utilized SHAP to interpret the output of machine learning models to screen out important features^32–34^. SHAP is considered a state-of-the-art machine learning interpreter^35^. The magnitude and direction of the influence of features on the output variables can be assessed by calculating SHAP values.

Here, we developed a LUAD biomarker potential scoring algorithm based on a novel deep learning framework, variational autoencoder joint multilayer perceptron (VAEjMLP), which combined the feature dimensionality reduction and classification prediction tasks and efficiently evaluated the importance of each feature gene by applying SHAP. This work successfully identified 19 LUAD biomarkers (LABs) and constructed a LUAD prognostic risk model. The functions mediated by the biomarkers in LUAD were investigated. The metabolic and immune landscapes of the prognostic risk model and its associations with angiogenesis, epithelial-mesenchymal transition (EMT), tumorigenic cytokines, and inflammation were explored. Our study provided reliable biomarkers for LUAD and may help to more accurately determine the survival of LUAD patients.

## Materials and methods

### LUAD data sources and preprocessing

The RNA-seq expression data and clinical information for LUAD were retrieved from The Cancer Genome Atlas (TCGA) (https://tcga-data.nci.nih.gov/tcga/) data portal. In constructing the prognostic risk model, the samples were filtered by clinical information including age, sex, survival time, overall survival (OS) status, pathologic T, N, M, stage, and history of previous cancer diagnosis, and a total of 482 patients with LUAD were included in the study. The GSE72094 derived from The Gene Expression Omnibus (GEO)^36^ (http://www.ncbi.nlm.nih.gov/geo) database was used as a validation set for the prognostic risk model, in which 393 LUAD samples with complete clinical information were included in this study. Single-cell RNA sequencing (scRNA-seq) data for 11 cases of distal normal lung tissue and 15 cases of primary LUAD were acquired from the GEO database, with accession number GSE131907. The GSE131907 dataset was used to analyze the expression of prognostic risk factors at the single-cell transcriptome level.

### Methods

**Figure 1:**
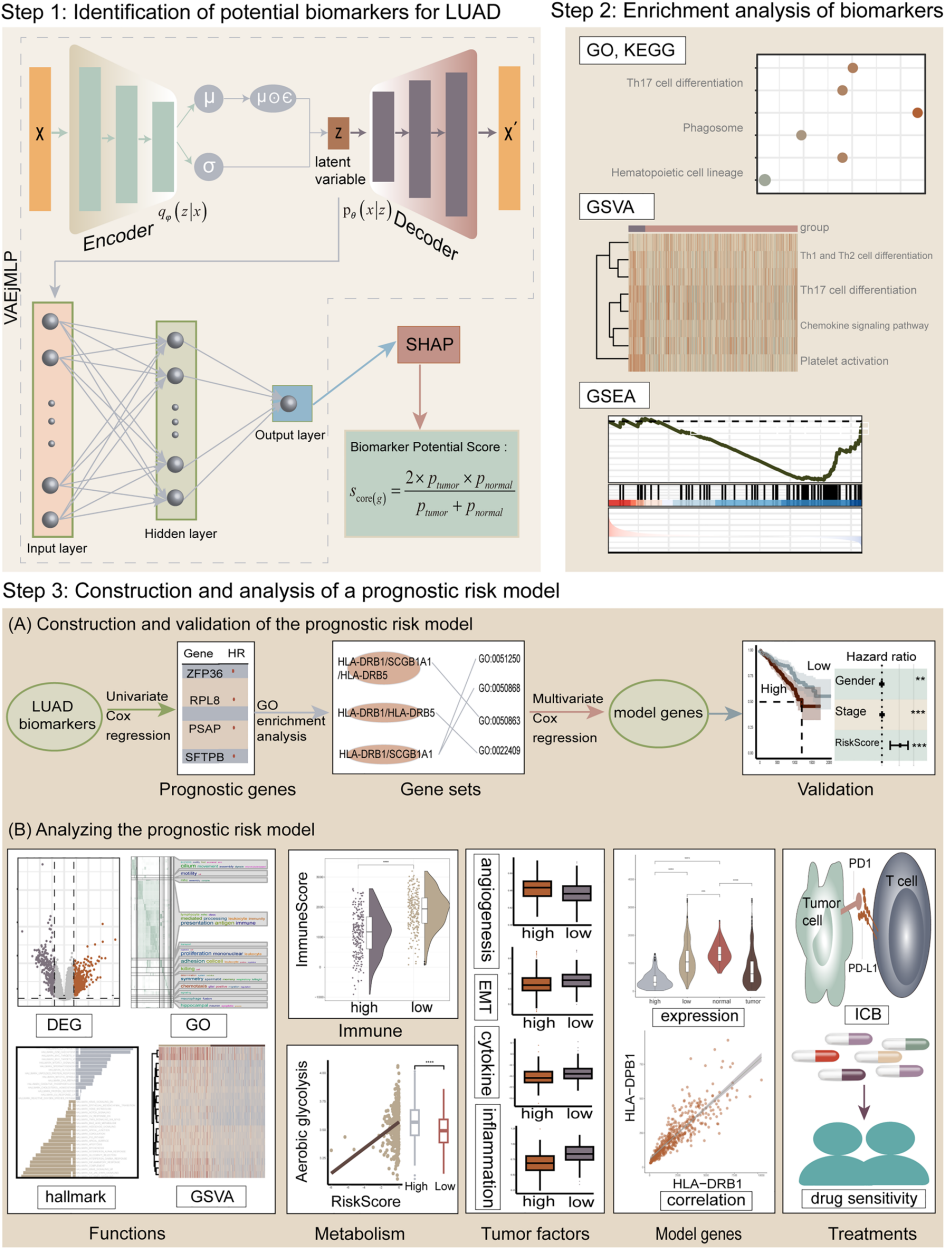
Flowchart of this study. Step 1, Constructing a joint framework VAEjMLP and then using the output of VAEjMLP as the input to Shapley Additive Explanations (SHAP), based on which a biomarker potential scoring algorithm was developed. Step 2, Enrichment analysis of biomarkers. Step 3, Construct a prognostic risk model for Lung adenocarcinoma (LUAD) by examining the prognostic significance and biological functions of the potential biomarkers, and analyze the model in multiple aspects. “DEG” means differentially expressed gene and “ICB” means immune checkpoint blockade.

Identifying reliable biomarkers is crucial for improving the prognosis of LUAD. Deep learning has been widely used in the exploration of biological problems. In this study, we proposed a novel deep learning framework based on VAE, MLP, and SHAP to identify biomarkers and construct a prognostic risk model for LUAD (the workflow of our investigation is shown in Fig. 1).

### LUAD biomarker identification framework

In order to mine biomarkers for LUAD, it is first necessary to identify genes that are highly altered between cancer and normal states. Based on the TCGA LUAD expression data consisting of read counts, the differentially expressed genes between normal and cancer samples were identified using the negative binomial distribution model of the R package “edgeR” (version 3.40.2), with a threshold set at | log2(fold change) |> 1 and *p*-value < 0.05^37^. To identify more stable differentially expressed genes, random sampling was performed on normal and cancer samples in a 1:6 ratio for 1000 times, where all normal samples were taken in each sampling. Differential expression analysis was conducted on each of the 1000 sets of samples and then the frequency of differential expression of each gene was counted. All differentially expressed genes, as well as genes with a frequency not less than 950 or 750, were selected as subsequent input features, respectively. The joint framework VAEjMLP was developed by combining the VAE model with MLP to perform the feature dimensionality reduction and classification prediction tasks simultaneously, allowing efficient representation learning with the help of the classification task. VAE consists of an encoder and a decoder and is an important generative model for dealing with feature representation capabilities. In this study, the encoder of the VAE is an inference model modelled by a neural network with the variational parameter φ as a learnable parameter, defined as an approximate posterior distribution, which is further assumed to be a Gaussian distribution:

1 $$\begin{array}{c}z=encoder\left(x\right) \sim {q}_{\varphi }\left(z|x\right)\end{array}$$

where $z$ is the latent variable and $x$ is the original input. The encoder will output the mean, $\mu$, and variance, $\sigma$, of the latent distribution, allowing for sampling from the hidden variable space. Since this operation of sampling is non-differentiable, to make the model trainable, sampling was performed by re-parameterization:

2 $$\begin{array}{c}z=\mu +\sigma \epsilon \end{array}$$

where $\epsilon \sim N\left(\mathrm{0,1}\right)$. The decoder still modelled by a neural network uses $z$ as input to reconstruct the original data to generate ${x}^{\mathrm{^{\prime}}}$:

3 $$\begin{array}{c}{x}^{\prime}=decoder\left(x\right)\sim {p}_{\theta }\left(x|z\right)\end{array}$$

where $\theta$ is the learnable parameter of the decoder and ${p}_{\theta }\left(x|z\right)$ follows a Bernoulli distribution. The objective function of the VAE is given by:

4 $$\begin{array}{c}{L}_{VAE}=BCE\left(x,{x}^{\prime}\right)+{D}_{KL}\left(N\left(\mu ,\sigma \right)\Vert N\left(\mathrm{0,1}\right)\right)\end{array}$$

where BCE is the binary cross entropy used to compute the distance between the original input $x$ and the reconstructed data ${x}{\prime}$, and ${D}_{KL}$ is the difference between the learned distribution and the standard Gaussian distribution. The VAE maps the original input to a low-dimensional latent space, which achieves feature dimensionality reduction. The latent space representation was then used as an input to the MLP to perform the classification task. The MLP adopted the cross entropy as a loss function, which can be expressed as:

5 $$\begin{array}{c}{L}_{MLP}=CE\left(y,{y}^{\prime}\right)\end{array}$$

where $y$ is the true label and ${y}^{\prime}$ is the predicted label. Ultimately, the loss function of the VAEjMLP can be expressed as:

6 $$\begin{array}{c}{L}_{VAE-MLP}=\lambda {L}_{VAE}+{L}_{MLP}\end{array}$$

where $\lambda$ is the hyperparameter that balances the training weights of VAE and MLP and was set to 0.001. The training objective of VAEjMLP is namely to minimize the loss function ${L}_{VAE-MLP}$. To train the VAEjMLP, we had randomly split the sample into an 80% training set and a 20% test set. The trained VAEjMLP was then passed into SHAP to quantify the contribution of each feature to the prediction results. SHAP interprets the predicted value ${y}^{\left(i\right)}$ of sample ${x}^{\left(i\right)}$ as the sum of the attributions of each feature in the sample, where the attributions are the SHAP values, which satisfy the following formula:

7 $$\begin{array}{c}{{\text{y}}}^{\left({\text{i}}\right)}={{\text{y}}}^{{\text{base}}}+\sum_{{\text{j}}=1}^{{\text{N}}}{\text{f}}\left({{\text{x}}}_{{\text{j}}}^{\left({\text{i}}\right)}\right)\end{array}$$

where ${y}^{base}$ is the model's baseline, which is the mean prediction of all training samples, $N$ represents the number of feature genes, and $f\left({x}_{j}^{\left(i\right)}\right)$ is the SHAP value of the j-th feature of the sample ${x}^{\left(i\right)}$. $f\left({x}_{j}^{\left(i\right)}\right)>0$ indicates that the feature is a positive contributor to the prediction, and vice versa, indicating that the feature is a negative contributor to the prediction. SHAP outputs the contribution rate of each feature gene in each sample. Based on the positive or negative impact of feature genes on the predicted output, this study proposed a biomarker potential scoring algorithm to assess the potential of the feature genes as markers for LUAD. First, the distribution of the data formed by all contribution rate values for all feature genes was examined, and a threshold for significant contribution was determined based on the distribution. In this study, a positive contribution rate indicates that the feature gene tends to classify the sample as a cancer sample and, conversely, a negative contribution rate when classified as a normal sample. For each feature gene $g$ with significant contribution, there exists the number of times ${C}_{P}$ counted as a significant positive contribution in cancer samples and the number of times ${C}_{N}$ counted as a significant negative contribution in normal samples. The number of cancer samples is ${N}_{tumor}$ and the number of normal samples is ${N}_{normal}$, then the proportion of feature gene $g$ that make a significant positive contribution to cancer samples is ${P}_{tumor}=\frac{{C}_{P}}{{N}_{tumor}}$, and the proportion of feature gene $g$ that make a significant negative contribution to normal samples is ${P}_{normal}=\frac{{C}_{N}}{{N}_{normal}}$. Then, the biomarker potential score ${S}_{core}\left(g\right)$ for feature gene $g$ is calculated as follows,

8 $$\begin{array}{c}{S}_{core}\left(g\right)=\frac{2\times {P}_{tumor}\times {P}_{normal}}{{P}_{tumor}+{P}_{normal}}\end{array}$$

The biomarker potential scores of all feature genes with significant contributions were calculated, and the 25th percentile of all scores in descending order was used as the lower bound to screen for potential LABs. The framework for identifying LABs was implemented in python using the PyTorch SHAP software packages^38,39^.

### Enrichment analysis of LABs

Gene Ontology (GO) and Kyoto Encyclopedia of Genes and Genomes (KEGG) enrichment analysis was performed using the R package “clusterProfiler”^40^. Biological pathways of target mitochondrial genes were annotated by MitoCarta 3.0 (http://www.broadinstitute.org/mitocarta)^41^ database. Gene set variation analysis (GSVA) is an unsupervised method that calculates enrichment scores for specific gene sets in each sample^42^. We used the GSVA to assess and compare differences in enrichment of functional gene sets across samples, with reference gene sets from the KEGG database resource (http://www.genome.jp/kegg/)^43^. Gene set enrichment analysis (GSEA) is used to assess the distribution trend of genes in a predefined gene set in a gene list ranked by phenotypic relatedness to determine their contribution to the phenotype^44^. In this study, GSEA was applied to identify biological functions associated with LUAD. | NES (normalized enrichment score) | > 1, Permutation test *p*-value < 0.05 and false discovery rate (FDR) < 0.25 were considered statistically significant.

### Constructing a prognostic risk model for LUAD

Univariate Cox regression analysis was performed on each LABs to screen genes significantly associated with OS in LUAD. Then, the screened genes were further filtered for biological functions and the regression coefficients of the genes were calculated using multivariate Cox regression analysis. A prognostic risk score was generated for each patient using the following formula,

9 $$\begin{array}{c}RiskScore=\sum_{i=1}^{M}Coe{f}_{Gene\left(i\right)}\times Ex{p}_{Gene\left(i\right)}\end{array}$$

where $M$ represents the number of samples, $Coe{f}_{Gene\left(i\right)}$ represents the regression coefficient of $Gene\left(i\right)$ and $Ex{p}_{Gene\left(i\right)}$ represents the expression level of $Gene\left(i\right)$. The samples were divided into high-risk and low-risk groups based on the median risk score.

### Enrichment analysis of the high- and low-risk groups

GO enrichment analysis was performed for differentially expressed genes between the high- and low-risk groups. Functional differences between the high- and low-risk groups were further analyzed by calculating enrichment scores for the hallmark and immunologic gene sets between the two groups using the GSVA method. The hallmark gene sets and immunologic signature gene sets were obtained from “h.all.v2023.1” and “c7.immunesigdb.v2023.1” of The Molecular Signatures Database (MSigDB) (https://www.gsea-msigdb.org/gsea/msigdb)^44^, respectively.

### Characterization of immune, metabolic, and other tumor-related factors

The relative infiltration abundance of 22 immune cell types in each sample was estimated based on gene expression data using the CIBERSORTx^45^ online tool. The R package “estimate” calculated stromal and immune scores to estimate the abundance of stromal cells and the level of immune cell infiltration in the sample's tumor microenvironment (TME) and combined the two scores to infer tumor purity^46^. Metabolic reprogramming is recognized as one of the key features of malignant tumors, with reprogramming of glycolysis, amino acid metabolism and lipid metabolism providing a tremendous energy demand for cancer cell proliferation^47^. Angiogenesis, EMT, tumorigenic cytokines and inflammation play crucial roles in tumor growth and progression^48–51^. Angiogenesis, EMT and tumorigenic cytokines-related genes were collected from the published literature of Qiu et al.^52^. Genes related to aerobic glycolysis, metabolism of glutamine, serine, glycine, arginine, methionine, tryptophan, fatty acids and sphingolipids, and inflammatory response were downloaded from MSigDB (https://www.gsea-msigdb.org/gsea/msigdb) database. Reference collections include “h.all.v2023.1”, “c2.cp.wikipathways.v2023.1”, “c2.cp.reactome.v2023.1”, “c2.cp.kegg.v2023.1” and “c5.go.bp.v2023.1”. Metabolic reprogramming, angiogenic activity, EMT, tumorigenic cytokines and inflammatory response scores were calculated for each sample by single-sample gene set enrichment analysis (ssGSEA) using the R package “GSVA”^42^.

### Prediction of immunotherapy response

The R package “EaSIeR” was used to predict the likelihood of patient response to immune checkpoint inhibitors (ICI). “EaSIeR” employs five types of features describing the immune TME to construct a model and uses transcriptome-based immune response scores (cytolytic activity (CYT), Roh immune score (Roh_IS), chemokines, Davoli immune signature (Davoli_IS), IFNy signature (IFNy), Expanded immune signature (Ayers_expIS), T-cell inflamed signature (Tcell_inflamed), T-cell inflamed signature (RIR), Tertiary lymphoid structures signature (TLS)) as learning targets to predict patients' ICI response^53^. Higher scores indicate a higher likelihood that a patient will respond to ICI therapy.

### Analysis of prognostic risk factors

The expression of prognostic risk factors and their correlations with immune-related genes and immune checkpoints were analyzed. Immune-related genes were downloaded from the TISIDB (http://cis.Hku.hk/TISIDB/) database^54^ and 79 immune checkpoints were from Hu et al.^55^.

### Statistical analysis

All statistical analyses were performed based on R software (4.2.1). Kolmogorov-Smirnov test was used for testing normality. Two-tailed Student's t-test was used to estimate the difference between the two groups when the data obeyed normal distribution, otherwise the Wilcoxon rank-sum test was performed. Spearman's rank correlation analysis was employed to explore the correlation between the variables. If not mentioned, *p* < 0.05 was considered statistically significant. If not specifically labelled, for the symbolic marking of statistical significance, we used the following convention, “ns”: *p* > 0.05; “*”: *p* <= 0.05; “** “: *p* <= 0.01; “***”: *p* <= 0.001; “****”: *p* <= 0.0001.

## Results

### Potential biomarkers for LUAD

**Figure 2:**
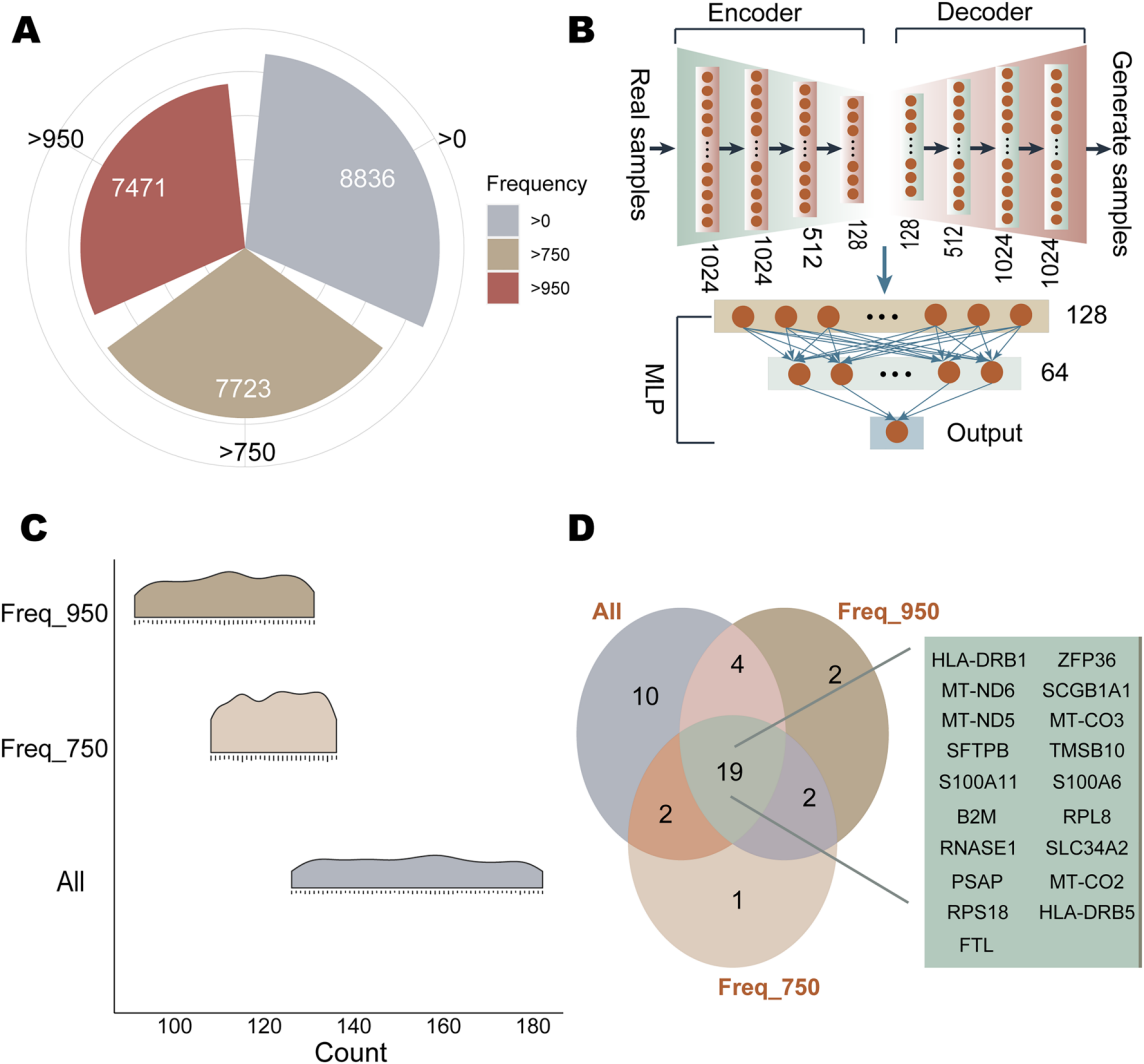
Potential biomarkers for LUAD. (**A**) Number of input features for the 3 sets. (**B**) Number of neurons in each hidden layer of VAEjMLP. (**C**) Number of candidate genes identified in each of the 1000 sample sets corresponding to the three sets of input features. Where “All”, “Freq_750”, “Freq_950” represent three sets of input features respectively. (**D**) The identified biomarkers for LUAD.

The TCGA database contains 539 cancer samples and 59 normal samples corresponding to LUAD. To identify stable differentially expressed genes, 1000 random samplings were performed between normal and cancer samples in a ratio of 1:6 (59:354). A total of 8836 differentially expressed genes were screened in the 1000 sample sets. Among them, 80.24% of the genes were differentially expressed with a frequency of 1000, 7471 genes had a frequency not less than 950, and 7723 genes had a frequency not less than 750 (Fig. 2A). For each of the above 1000 sets of samples, three groups of differentially expressed genes screened according to frequency thresholds were respectively used as feature inputs for VAEjMLP. For the VAEjMLP model, it used a VAE model consisting of an encoder and decoder modeled by a neural network, respectively, to achieve feature dimensionality reduction, and input the reduced features into an artificial neural network MLP for classification prediction. In VAEjMLP, the encoder part of VAE consisted of four hidden layers, with 1024 neurons in hidden layer 1 and hidden layer 2, 512 in hidden layer 3, and 128 in hidden layer 4, and the decoder had a mirrored structure with the encoder (Fig. 2B). The latent vector of dimension 128 obtained from VAE training was passed into a three-layer MLP with 128 neurons in the first layer, 64 in the second layer and the third layer as the output layer (Fig. 2B). Both VAE and MLP used the Leaky ReLU activation function. The number of epochs was set to 250, with the first 50 epochs for unsupervised training of VAE, the 51st–100th epochs for classification training of MLP, and the 101st–250th epochs for joint training of VAEjMLP. The area under the curve (AUC) of the trained VAEjMLP model reached over 0.999, and the accuracy, precision, and recall all reached over 0.99, 0.955, and 0.994, respectively. For each set of input samples, three groups of significant contributing genes were screened using ±5%, ±1%, and ±0.5% of the distribution formed by all the contribution values of the SHAP output as thresholds, respectively. Using the biomarker potential scoring algorithm, each significant contributing gene received its scores in each set of samples. The quartiles of the descending biomarker potential scores of the three groups of significantly contributing genes were used as lower bounds to screen the genes, respectively, and the intersection was considered as candidates. When all differentially expressed genes, genes with a frequency not less than 750, and genes with a frequency not less than 950 were used as features, respectively, the number of candidate genes identified in 1000 sample sets was 127–183, 109–137, and 92–132, respectively (Fig. 2C). Eventually, the 19 shared genes of all candidates were identified as potential LABs (Fig. 2D).

### Association analysis of LABs with LUAD

**Figure 3:**
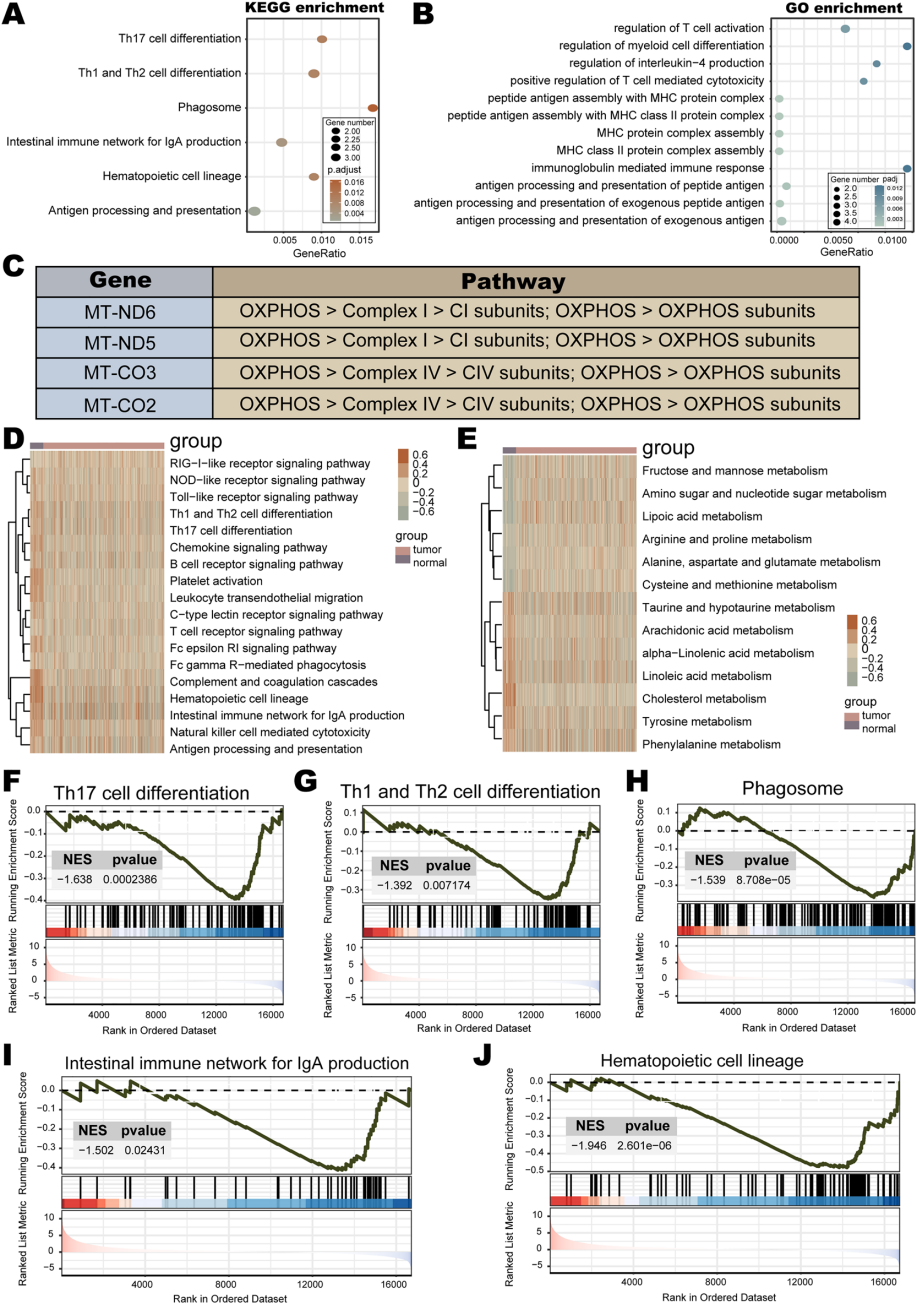
Association analysis of LUAD biomarkers (LABs) with LUAD. (**A**) KEGG, and (**B**) GO enrichment analysis of LABs. (**C**) Pathway annotation of mitochondrial genes. (**D**–**E**) GSVA analyses in TCGA LUAD. (**F**–**J**) GSEA analyses based on TCGA LUAD.

LABs consist of 15 nuclear and 4 mitochondrial genes. The biological functions of nuclear genes were explored using GO and KEGG enrichment analysis. The results demonstrated that nuclear genes were mainly enriched in immune pathways, such as MHC protein complex assembly, Th1 and Th2 cell differentiation, antigen processing and presentation, regulation of T cell activation, etc. (Fig. 3A,B). In addition, four mitochondrial genes (*MT-ND6*, *MT-ND5*, *MT-CO3*, *MT-CO2*) were all involved in driving mitochondrial oxidative phosphorylation (OXPHOS) (Fig. 3C). The OXPHOS system serves as the center of cellular metabolism and is critical for energy production in eukaryotic cells^56^. Besides, the GSVA results showed that immune pathways, such as platelet activation, leukocyte transendothelial migration, natural killer cell mediated cytotoxicity, and Th1 and Th2 cell differentiation, were mostly enriched in the normal group as compared to the LUAD group (Fig. 3D). Simultaneously, the normal and LUAD groups presented significant differences in the enrichment of metabolic pathways (e.g., fructose and mannose metabolism, arginine and proline metabolism, and cholesterol metabolism) (Fig. 3E). The GSEA was also employed to evaluate the signaling pathways involved in nuclear genes. The results indicated that nuclear genes of LABs were negatively linked to immune pathways (Th17 cell differentiation, Th1 and Th2 cell differentiation, Phagosome, intestinal immune network for IgA production, and hematopoietic cell lineage) in LUAD (Fig. 3F–J). These results illustrated that LABs were implicated in the regulation of immune and metabolic functions in LUAD.

### Construction and validation of a prognostic risk model

**Figure 4:**
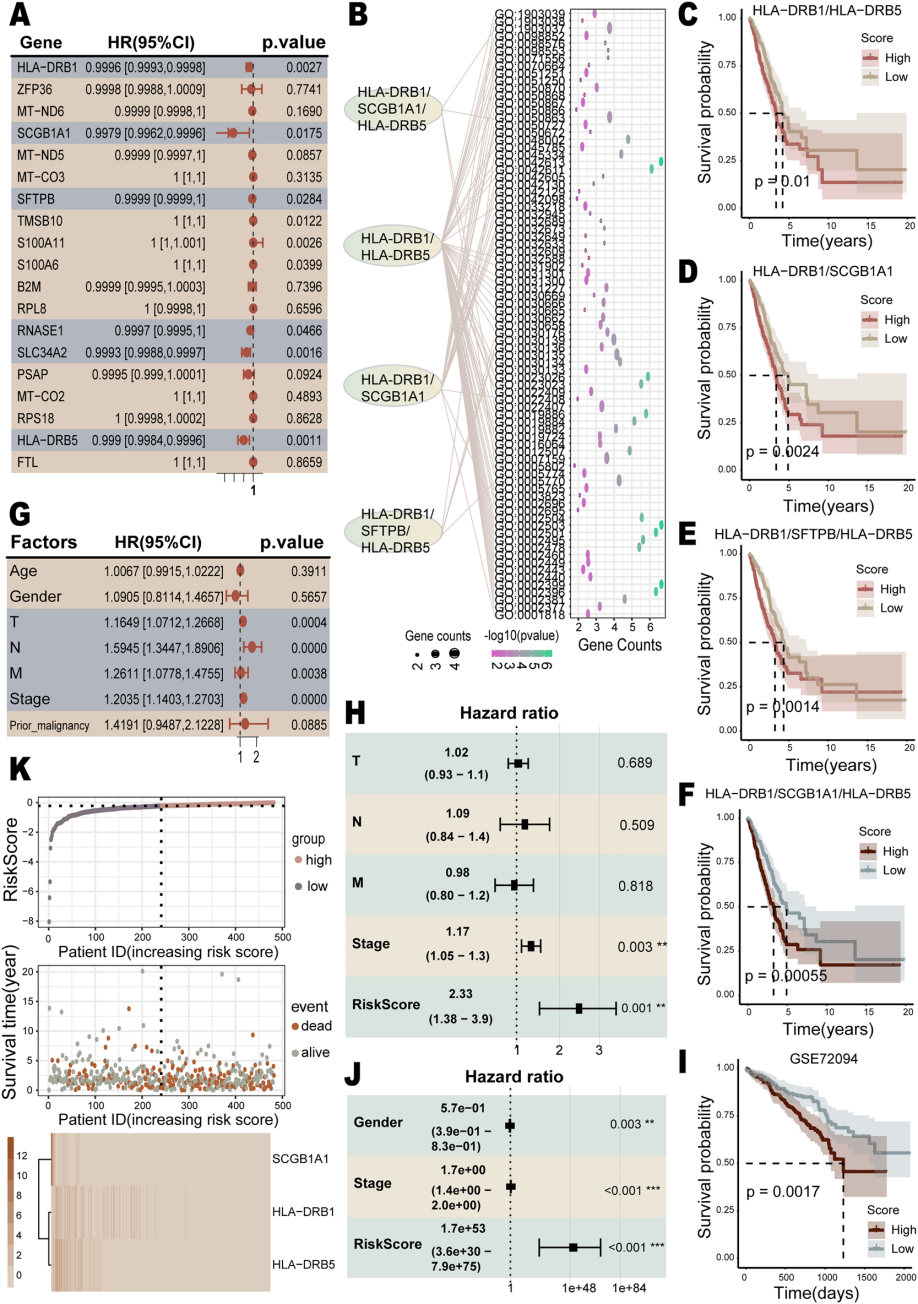
The construction of the prognostic risk model. (**A**) Forest plot of univariate Cox regression analysis for the LABs. Genes with *p*-value < 0.05 are highlighted with a grayish blue background. (**B**) Enriched for genes with the same GO entry. (**C**–**F**) Kaplan–Meier survival curve analysis for the gene sets *HLA-DRB1*/*HLA-DRB5*, *HLA-DRB1*/*SCGB1A1*, *HLA-DRB1*/*SFTPB*/*HLA-DRB5*, and *HLA-DRB1*/*SCGB1A1*/*HLA-DRB5*. (**G**) Forest plot of univariate Cox regression analysis for clinical environmental factors. Factors with *p*-value < 0.05 are highlighted with a grayish blue background. (**H**) Forest plot of multivariate Cox regression analysis of the risk score combined with survival-related clinical factors. (**I**) Kaplan–Meier survival curve analysis of the validation set. (**J**) Multivariate Cox regression analysis of the validation set. (**K**) Upper panel: risk scores of patients in ascending order. The horizontal dashed line represents the median risk score. Middle panel: survival time of patients in ascending order of risk score. Bottom panel: heatmap of gene expression levels in patients sorted by ascending risk score.

The univariate Cox regression analysis showed that *HLA-DRB1*, *SCGB1A1*, *SFTPB*, *RNASE1*, *SLC34A2*, and *HLA-DRB5* among the LABs were significantly associated with LUAD survival (Fig. 4A). Further analysis was conducted using survival-related biomarker sets that were enriched in the same GO entries. There were four sets of genes enriched in the same GO entries (Fig. 4B): *HLA-DRB1*/*HLA-DRB5*, *HLA-DRB1*/*SCGB1A1*/*HLA-DRB5*, *HLA-DRB1*/*SCGB1A1*, and *HLA-DRB1*/*SFTPB*/*HLA-DRB5*. The Kaplan-Meier survival curve analysis for all four gene sets showed significant associations with LUAD survival. More specifically, the significant *p*-values for the *HLA-DRB1*/*HLA-DRB5* (Fig. 4C), *HLA-DRB1*/*SCGB1A1* (Fig. 4D), and *HLA-DRB1*/*SFTPB*/*HLA-DRB5* (Fig. 4E) sets were 0.01, 0.0024, and 0.0014, respectively. The *HLA-DRB1*/*SCGB1A1*/*HLA-DRB5* set presented the most significant difference in survival rates (*p*-value=0.00055) (Fig. 4F). Ultimately, the set *HLA-DRB1*/*SCGB1A1*/*HLA-DRB5*, which was most correlated with LUAD survival, was selected to construct the LUAD prognostic risk model and risk scores for patients were calculated. The regression coefficients for *HLA-DRB1*, *SCGB1A1*, and *HLA-DRB5* in the LUAD prognostic risk model were  − 7.36e−05,  − 1.51e−03, and  − 7.43e−04, respectively. It can be observed that the patients with higher risk scores had poorer OS (Fig. 4F). The univariate Cox regression analysis suggested that pathological T, N, M and stage staging were significantly associated with survival in LUAD patients (Fig. 4G). A multivariate Cox regression analysis was performed to explore the predictive independence of the prognostic risk model by combining these survival-related clinicopathological factors with the risk score. The results demonstrated that the risk score (HR = 2.33, 95% CI = 1.38–3.9, *P* = 1e−3) served as an independent risk factor for OS in patients with LUAD (Fig. 4H). The GSE72094 dataset derived from the GEO database validated the accuracy of the prognostic risk model (Fig. 4I,J). The association of the risk score with survival status and prognostic risk factors was further explored (Fig. 4K). Overall, a higher risk score was associated with a higher number of deaths, indicating a poorer prognosis and higher risk of death in the high-risk group. Compared to the low-risk group, the prognostic risk factors *SCGB1A1*, *HLA-DRB1*, *HLA-DRB5* were all down-regulated in the high-risk group.

### Enrichment analysis of the prognostic risk model

**Figure 5:**
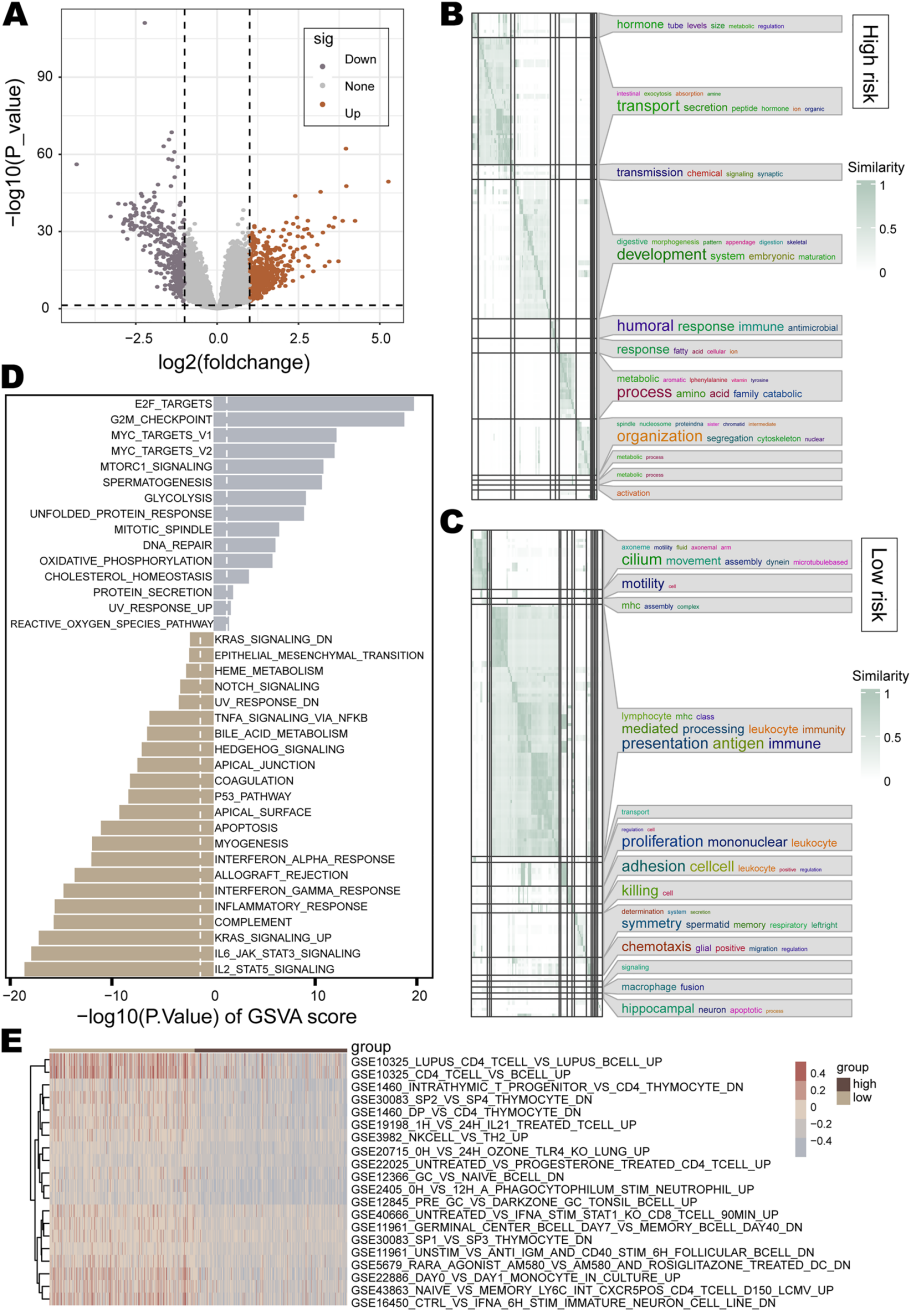
Enrichment analysis of the prognostic risk model. (**A**) Differentially expressed genes between the high- and low-risk groups. (**B**, **C**) Clustering maps of GO entries enriched by up-regulated genes in the high-risk and low-risk groups. Word cloud annotations are appended on the right, and font sizes reflect word frequencies. (**D**) Enrichment analysis of cancer hallmark gene sets with differential enrichment between high-risk and low-risk groups. Vertical dashed lines represent significance thresholds. (**E**) Heatmap of enrichment scores for immunologic signature gene sets differentially enriched in the high-risk and low-risk groups.

Genes differentially expressed in the high- and low-risk groups were identified, and 459 up-regulated genes and 352 down-regulated genes were recognized in the high-risk group (Fig. 5A). The up-regulated genes were mainly enriched in various metabolic pathways, secretion and transportation of substances, and pathways related to development (Fig. 5B). The down-regulated genes were predominantly enriched in various immune-related pathways (Fig. 5C). To further explore the differences in biological functions of different risk groups, we conducted GSVA analysis. Among the 50 cancer hallmark gene sets, significant enrichment differences were observed in 37 gene sets between the high and low-risk groups (Fig. 5D). Compared with the low-risk group, the high-risk group was mainly accompanied by the enrichment of cell proliferation pathways (such as E2F targets, G2M checkpoint, MYC targets, etc.) and metabolic pathways (such as MTORC1 signaling, glycolysis, oxidative phosphorylation, etc.) (Fig. 5D). Also of concern was the significant down-regulation of the activity of immune-related pathways in the high-risk group compared to the low-risk group, such as IL2/STAT5 signaling, IL6/JAK/STAT3 signaling, inflammatory response, and other pathways (Fig. 5D). Both GO and GSVA analyses illustrated that metabolism-related pathways were primarily enriched in the high-risk group and immune-related pathways were mostly enriched in the low-risk group. The characteristics of unlimited tumor proliferation often require alterations in energy metabolism^57^, and studies have indicated that increased expression of glycolytic enzymes correlates with poor prognosis in lung cancer patients^58,59^. The immune system plays a dual role of both promotion and inhibition of cancer development^60^, and the signaling pathways IL2/STAT5 and IL6/JAK/STAT3 were closely associated with the prognosis of pancreatic ductal adenocarcinoma and olfactory neuroblastoma^61,62^. The immunologic signature gene sets derived from the MSigDB database represent immune states and perturbations signatures. Therefore, we used GSVA analysis to compare the differences in the enrichment of immunologic signature gene sets between the high- and low-risk groups. Among the 4,872 immunologic signature gene sets, there were differences in enrichment in 3,574 gene sets between the two risk groups. Specifically, the high-risk group exhibited significantly lower enrichment scores in 2,585 immunologic signature gene sets compared to the low-risk group. Not only that, the top 100 gene sets with the most significant differences were all showed down-regulation of activity in the high-risk group (Fig. 5E presents the top 20 gene sets with the most significant differences).

### Immune landscapes for the prognostic risk model

**Figure 6:**
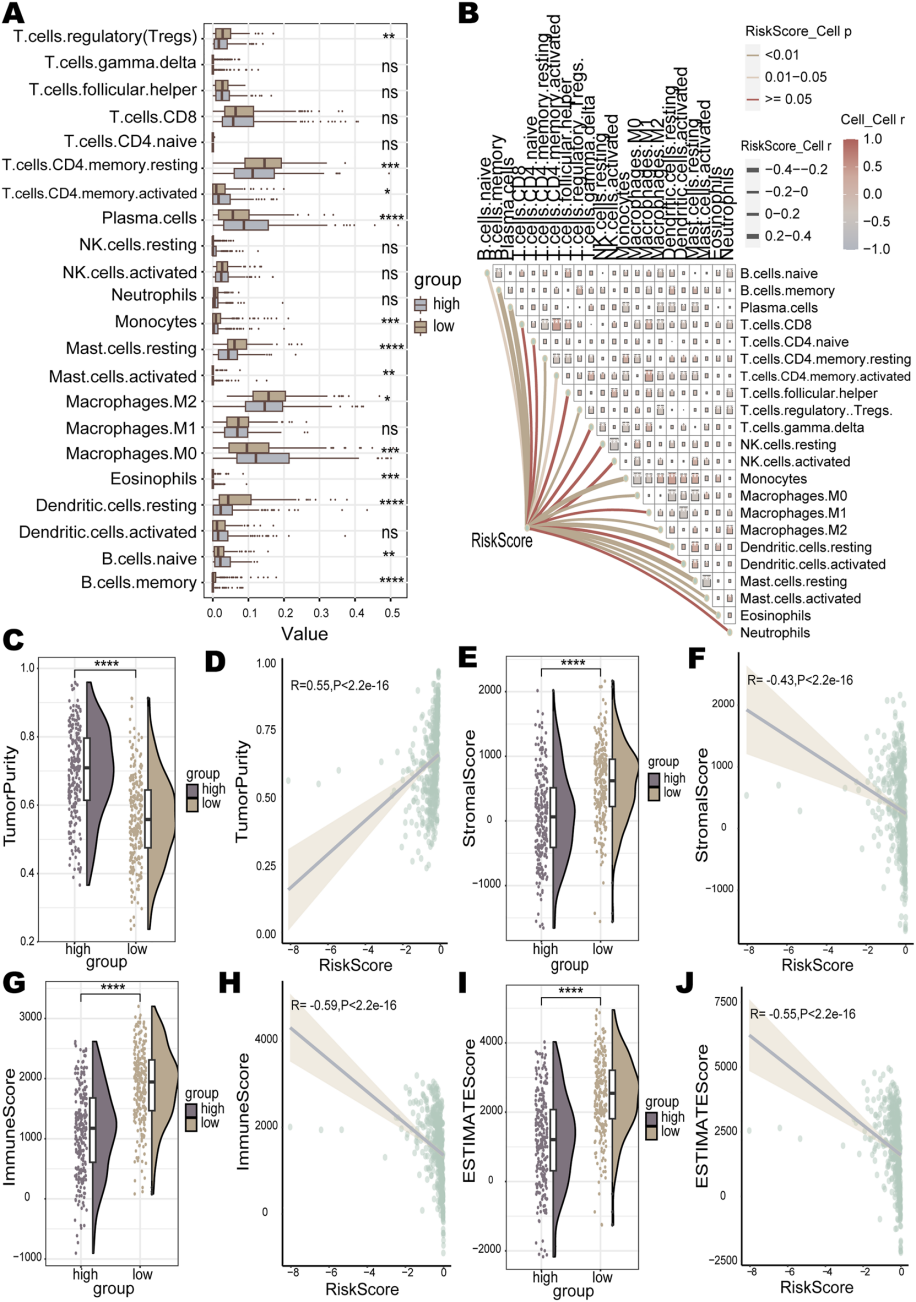
Immune landscape of the prognostic risk model. (**A**) Infiltration levels of 22 immune cells in high- and low-risk groups. (**B**) The correlation between risk scores and immune cell infiltration levels in patients. (**C**) Tumor purity, (**E**) stromal score, (**G**) immune score, and (**I**) ESTIMATE score in the high- and low- risk groups. Correlation of risk scores with (**D**) tumor purity, (**F**) stromal score, (**H**) immune score, and (**J**) ESTIMATE score.

The high-risk group presented significantly down-regulation of immune pathway activity compared to the low-risk group. Therefore, the CIBERSORT algorithm was employed to calculate the immune infiltration of the high-risk and low-risk groups, while the ESTIMATE algorithm was utilized to assess the immune TME in both groups. The level of infiltration of 22 immune cell types in LUAD patients was estimated using the CIBERSORT algorithm. The proportions of 13 immune cell types infiltrated showed significant differences between the two risk groups. The infiltration proportions of B cells naive, eosinophils, macrophages M0, mast cells activated, plasma cells, and T cells CD4 memory activated were higher in the high-risk group than in the low-risk group (Fig. 6A). The correlation between the risk score and the level of infiltration of these six immune cells was significant and positive (Fig. 6B). B cells memory, dendritic cells resting, macrophages M2, mast cells resting, monocytes, T cells CD4 memory resting, and T cells regulatory (Tregs) infiltration levels were higher in the low-risk group (Fig. 6A). The infiltration proportions of these 7 immune cell types showed a significant and negative correlation with the risk score (Fig. 6B). Several LUAD survival studies have demonstrated that macrophages M0, mast cells activated and T cells CD4 memory activated were accompanied by higher levels of infiltration in high-risk groups, and infiltration levels of B cells memory, dendritic cells resting, mast cells resting, monocytes, T cells CD4 memory resting and T cells regulatory (Tregs) were higher in low-risk groups^63–65^. This aligns with the findings of our study. Stromal scores for stromal cell abundance, immune scores for the extent of immune cell infiltration and tumor purity in the TME were estimated using ESTIMATE for different risk groups. The results indicated that patients in the high-risk group had higher tumor purity than those in the low-risk group (*p* < 2.2e−16) (Fig. 6C). The risk score was positively correlated with tumor purity in LUAD patients (R = 0.55, *p* < 2.2e−16) (Fig. 6D). Compared to the low-risk group, patients in the high-risk group had significantly lower stromal scores (*p* = 3.2e−16), immune scores (*p* < 2.2e−16), and ESTIMATE scores (*p* < 2.2e−16) (Fig. 6E,G,I). The risk score showed negative correlations with the stromal score (R = − 0.43, *p* < 2.2e−16), the immune score (R = − 0.59, *p* < 2.2e−16), and the ESTIMATE score (R = − 0.55, *p* < 2.2e−16) (Fig. 6F,H,J). Taken together, we thought that the immune response effect in the high-risk group may be inferior to that of the low-risk group. Multiple studies have separately constructed survival models for LUAD, and their findings consistently demonstrated that LUAD patients with high-risk scores exhibited lower immune scores, stromal scores, ESTIMATE scores, and higher tumor purity^64,66–68^. This provides valid support for our findings.

### Metabolism landscapes and tumor-related factors analysis for the prognostic risk model

**Figure 7:**
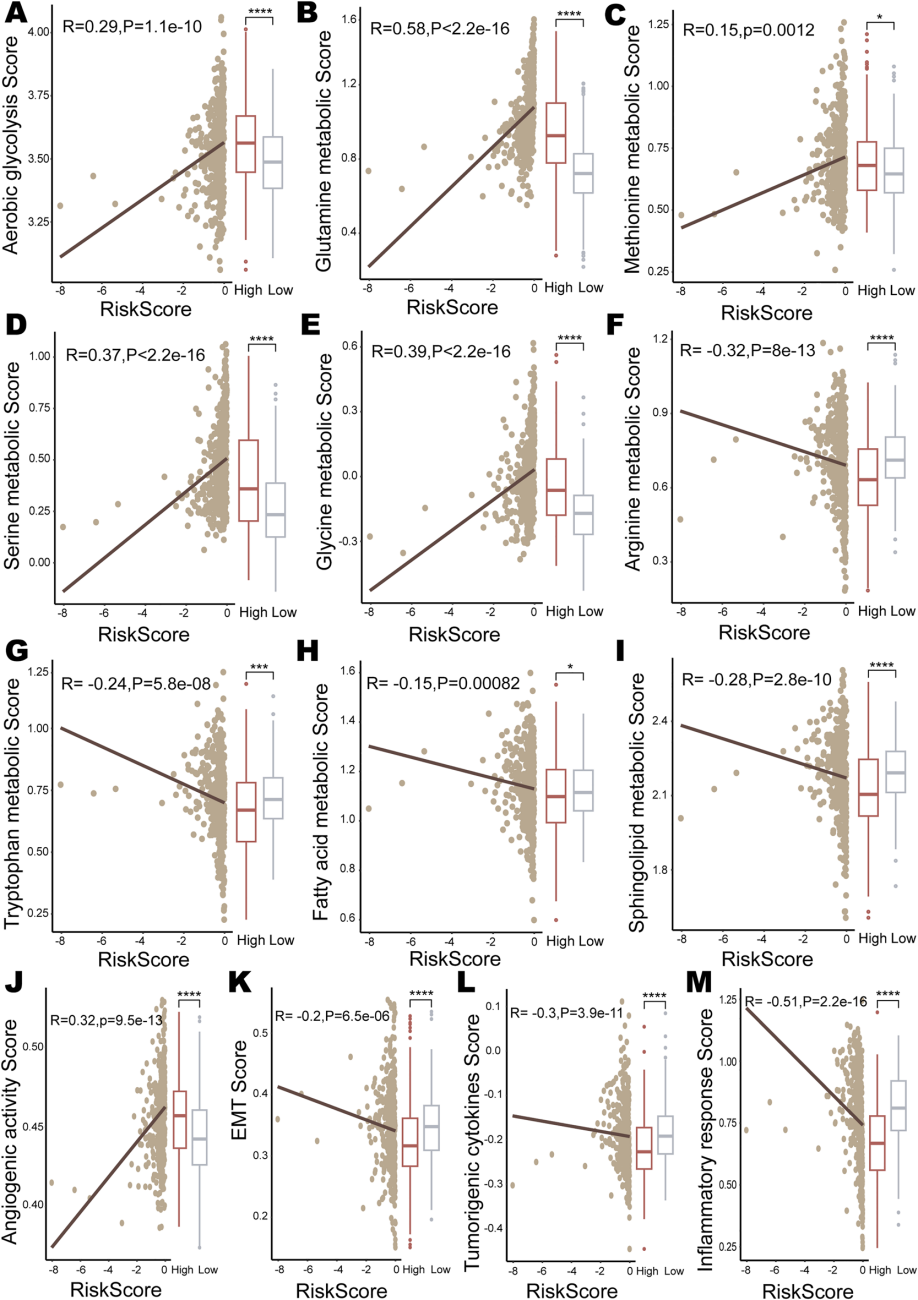
Metabolism landscapes and tumor-related factors analysis for the prognostic risk model. Correlations between risk scores and metabolic reprogramming scores for (**A**) aerobic glycolysis, (**B**) glutamine, (**C**) methionine, (**D**) serine, (**E**) glycine, (**F**) arginine, (**G**) tryptophan, (**H**) fatty acids, and (**I**) sphingolipids. The correlations between risk scores and scores for (**J**) angiogenesis activity, (**K**) EMT, (**L**) tumorigenic cytokines, and (**M**) inflammatory response. “High” and “Low” represent the high-risk and low-risk groups, respectively.

Cancer cells reprogram their metabolic pathways to meet the needs of tumor initiation and progression^69,70^. GO and GSVA analyses revealed significant metabolic differences between the high- and low-risk groups. The metabolic reprogramming differences between the high- and low-risk groups were investigated by calculating the reprogramming scores of glycolysis, amino acid metabolism, and lipid metabolism in LUAD patients. Aerobic glycolysis is a key metabolic hallmark of cancer, promoting the survival and proliferation of cancer cells. The preference of cancer cells for glycolysis is often associated with poorer clinical outcomes^71^. The risk score was positively correlated with the aerobic glycolysis reprogramming score (R = 0.29,* p* = 1.1e−10) (Fig. 7A). The patients in the high-risk group had significantly higher aerobic glycolysis reprogramming scores (*p* = 2.9e−06) than those in the low-risk group (Fig. 7A). Amino acid metabolism is one of the pivotal nutrients supporting cancer cell growth, and the metabolism of glutamine, methionine, serine, glycine, arginine, and tryptophan is deregulated in many cancers^72–76^. The risk score was positively correlated with the metabolic reprogramming scores of glutamine (R = 0.58, *p* < 2.2e−16) and methionine (R = 0.15, *p* = 1.2e−3) (Fig. 7B,C). The metabolic reprogramming scores of glutamine (*p* < 2.2e−16) and methionine (*p* = 2.4e−2) were both higher in the high-risk group than in the low-risk group (Fig. 7B,C). Studies have shown that the metabolism of glutamine and methionine not only drives tumor cell growth, but also creates a TME that benefits from tumor immune escape^77–79^. Meanwhile, significant positive correlations were observed between the risk score with the metabolic reprogramming scores of serine (R = 0.37, *p* < 2.2e−16) and glycine (R = 0.39, *p* < 2.2e−16) (Fig. 7D,E). The high-risk group presented higher metabolic reprogramming scores of serine (*p* = 3e−09) and glycine (*p* = 3.6e−13) (Fig. 7D,E). The upregulation of serine\glycine metabolism is associated with poor clinical outcomes in several cancers^80^. While the patients' metabolic reprogramming scores for arginine (R = − 0.32, *p* = 8e−13) and tryptophan (R = − 0.24, *p* = 5.8e−08) were negatively correlated with the risk scores (Fig. 7F,G). The metabolic reprogramming scores of arginine (*p* = 3.2e−08) and tryptophan (*p* = 1.3e−4) were lower in the high-risk group (Fig. 7F,G). Lipids are acting as central players in cancer biology^81^, and metabolic imbalances in fatty acids and sphingolipids assume a crucial role in cancer development^82,83^. There were significant negative correlations between the risk scores with the metabolic reprogramming scores of fatty acids (R = − 0.15, *p* = 8.2e−4) and sphingolipids (R = − 0.28, *p* = 2.8e−10) (Figure 7H,I). The patients with higher metabolic reprogramming scores for fatty acids (*p* = 5e−2) and sphingolipids (*p* = 8.9e−07) tended to be accompanied by lower risk scores (Fig. 7H,I).

In addition, angiogenesis, EMT, tumorigenic cytokines and inflammation play important and complex roles in the progression of malignant tumors. Thereby, angiogenic activity score, EMT score, tumorigenic cytokine score and inflammatory response score were evaluated for the prognostic risk model, respectively. The angiogenesis activity score in the high-risk group was significantly higher than that in the low-risk group (*p* = 2.9e−08) (Fig. 7J). There was a significant trend of positive correlation between the risk score and the angiogenesis activity score (R = 0.32, *p* = 9.5e−13) (Fig. 7J). Angiogenesis is associated with poor prognosis in several cancers, including NSCLC, and anti-angiogenic drugs are being developed^84,85^. Patients in the high-risk group had significantly lower EMT scores (*p* = 2e−05), tumorigenic cytokine scores (*p* = 9.4e−08) and inflammatory response scores (*p* < 2.2e−16) than those in the low-risk group (Fig. 7K–M). The risk score was negatively correlated with the EMT score (R = − 0.2, *p* = 6.5e−06), tumorigenic cytokine score (R = − 0.3, *p* = 3.9e−11), and inflammatory response score (R = − 0.51, *p* < 2.2e−16) (Fig. 7K–M).

In conclusion, patients with poor prognosis experienced significant changes in metabolic reprogramming, angiogenesis, EMT, tumorigenic cytokines, and inflammation.

### Characterization of prognostic risk factors

**Figure 8:**
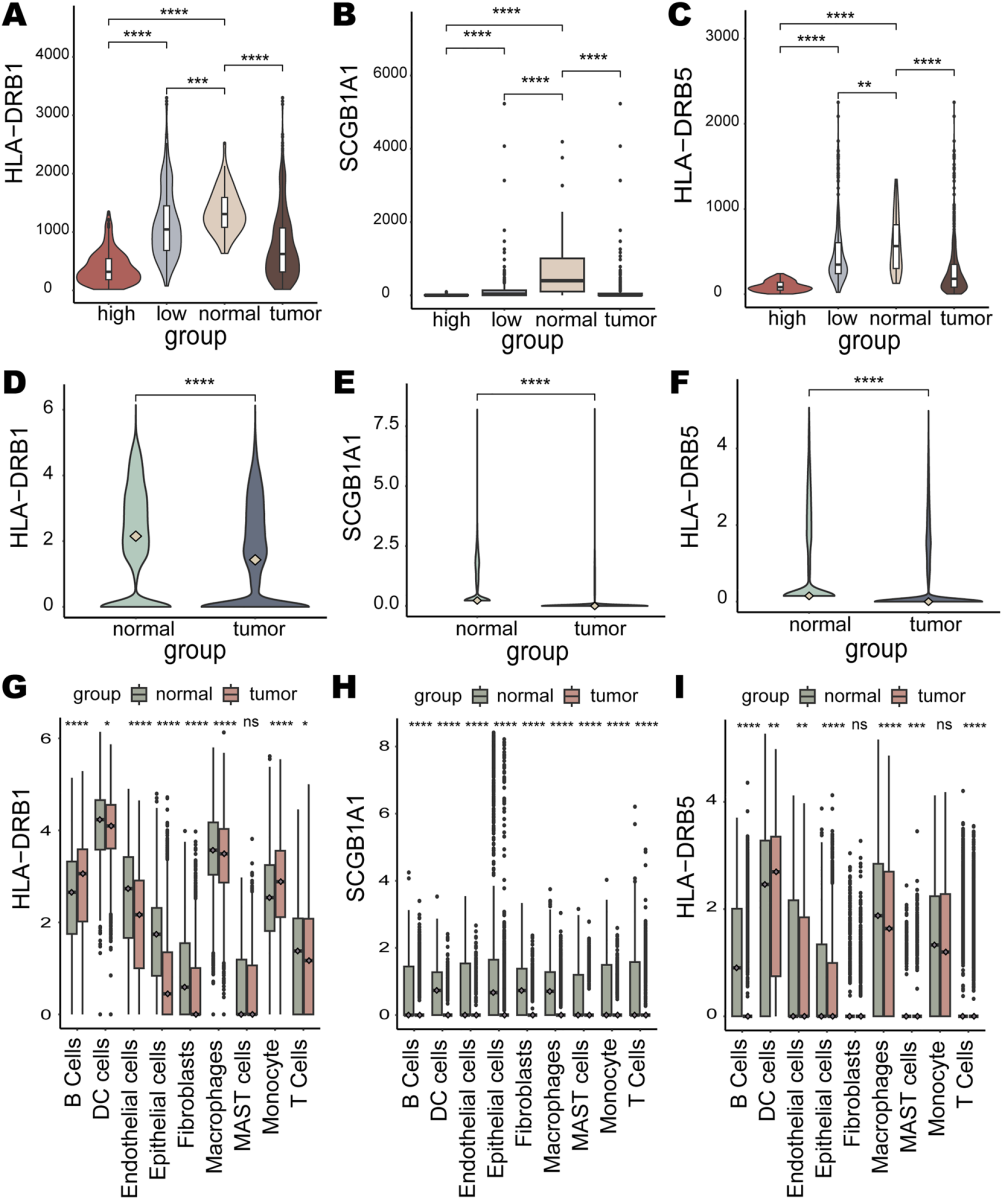
Characterization of the expression of prognostic risk factors. Expression of (**A**) *HLA-DRB1*, (**B**) *SCGB1A1*, and (**C**) *HLA-DRB5* in normal tissues, cancerous tissues and high- and low-risk groups. Overall expression of (**D**) *HLA-DRB1*, (**E**) *SCGB1A1*, and (**F**) *HLA-DRB5* at the single-cell level in normal and cancer states. Expression of (**G**) *HLA-DRB1*, (**H**) *SCGB1A1*, and (**I**) *HLA-DRB5* in various cell types in normal and cancer states.

We further analyzed the prognostic risk factors *HLA-DRB1*, *SCGB1A1* and *HLA-DRB5*. They were all significantly lower expressed in the high-risk group than in the low-risk group (Fig. 8A–C). *HLA-DRB1*, *SCGB1A1* and *HLA-DRB5* were accompanied by lower expression in LUAD tissues compared to their expression in normal lung tissues (Fig. 8A–C). The scRNA-seq dataset GSE131907 was used to explore the expression of prognostic risk factors in normal lung tissue and primary LUAD. With data preprocessing and quality control, a total of 92,951 single cells were captured and clustered into nine cell types including endothelial cells, epithelial cells, monocytes, etc. Similarly, the expression of prognostic risk factors was observed at the single-cell transcriptome level were all significantly lower in the disease state than in the normal state (Fig. 8D–F). Differential expression of prognostic risk factors was also compared in different cell types between normal and LUAD states. *HLA-DRB1* exhibited differential expression between normal and disease states in all cell types except MAST cells (Fig. 8G). In the LUAD state, *HLA-DRB1* expression was significantly higher in all cell types except B Cells and Monocyte than in the normal state (Fig. 8G). *SCGB1A1* expression was significantly higher in all cell types in the normal state than in the LUAD state (Fig. 8H). *HLA-DRB5* showed significant expression differences between normal and disease states in B cells, DC cells, endothelial cells, epithelial cells, macrophages, MAST cells, and T cells (Fig. 8I).

**Figure 9:**
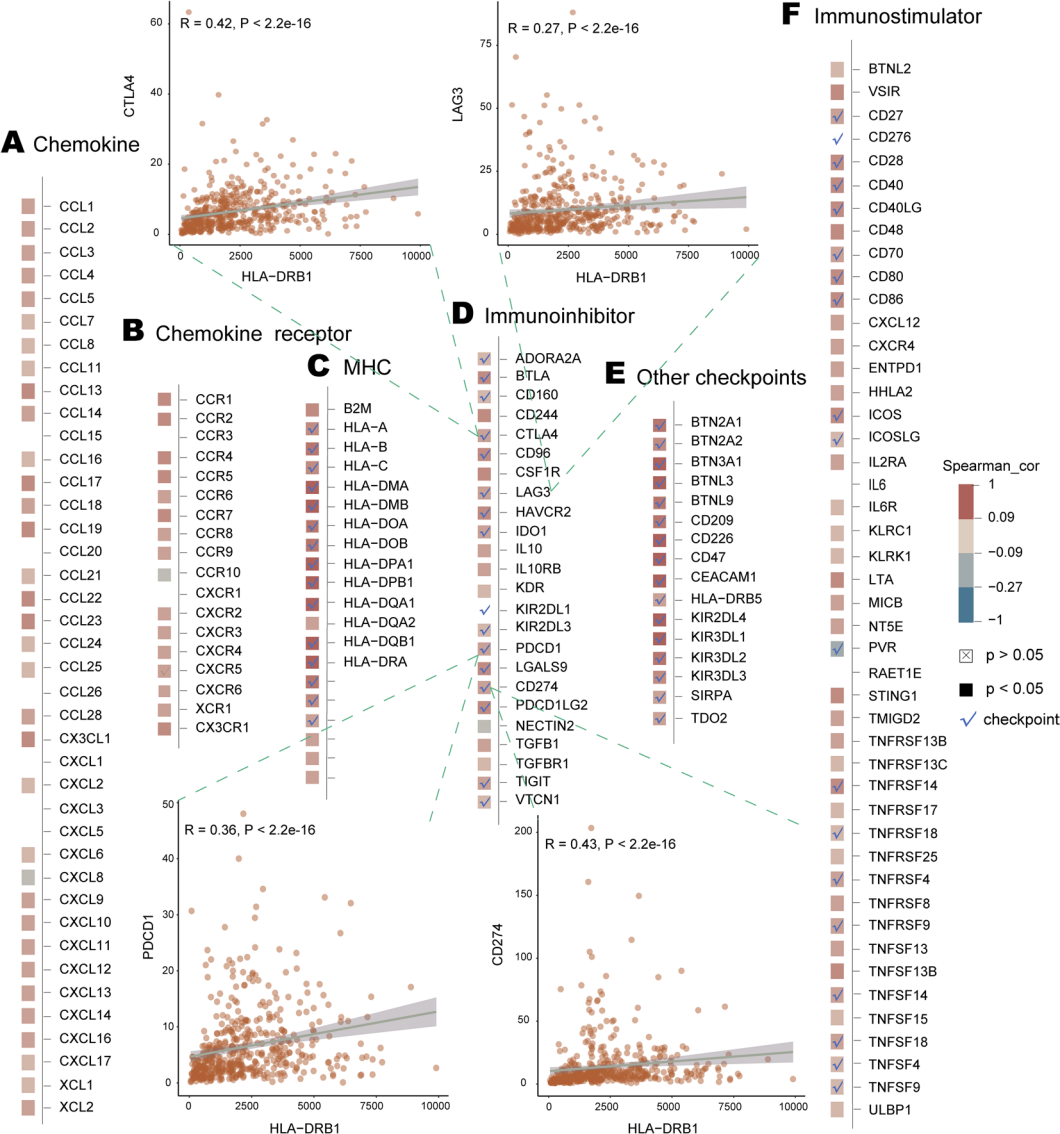
The correlation of *HLA-DRB1* with immune-related genes and immune checkpoints: (**A**) chemokine genes, (**B**) chemokine receptor genes, (**C**) MHC, (**D**) immunosuppressive genes, (**E**) other immune checkpoints, and (**F**) immunostimulatory genes. “other immune checkpoints” means immune checkpoints other than immune-related genes.

Recently, targeting TME has emerged as a promising method for cancer treatment^86^. Immune-related genes, as important components of TME, play a crucial role in tumorigenesis and TME homeostasis^87^. In parallel, immune checkpoints can modulate the degree of immune activation in TME^88^. Co-expression analysis was performed to explore the correlation of the prognostic risk factors *HLA-DRB1*, *SCGB1A1* and *HLA-DRB5* with immune-related genes (chemokines, chemokine receptors, MHC, immunostimulatory genes, immunosuppressive genes) and immune checkpoints. The prognostic risk factors *HLA-DRB1* and *HLA-DRB5* are immune checkpoints, of which *HLA-DRB1* is also an MHC gene. The prognostic risk factors *HLA-DRB1* (Fig. 9), *SCGB1A1* (Supplementary Fig. S1), and *HLA-DRB5* (Supplementary Fig. S2) were positively correlated with the majority of immune-related genes and immune checkpoints. PD-1 (PDCD1), PD-L1 (CD274), CTLA4 and LAG3 are common immune checkpoints focused on by researchers. *HLA-DRB1* was positively correlated with those four immune checkpoints (*PD-1*: R = 0.36, *p* = 2.22e−16; *PD-L1*: R = 0.43, *p* = 0; *CTLA4*: R = 0.42, *p* = 0; *LAG3*: R = 0.27, *p* = 1.13e−09), as was *HLA-DRB5* (*PD-1*: R = 0.30, *p* = 8.75e−12; *PD-L1*: R = 0.38, *p* = 0; *CTLA4*: R = 0.36, *p* = 8.88e−16; *LAG3*: R = 0.20, *p* = 1.44e−05), whereas *SCGB1A1* showed a positive correlation with *CTLA4* (R = 0.13, *p* = 4.6e−3). The findings of Aram et al.^89^ showed that HLA-DRB1 was associated with anti-PD-1/PD-L1 therapy response in NSCLC. A recent study demonstrated the ability of fucosylation of HLA-DRB1 to enhance the efficacy of immune checkpoint blockade anti-PD1 drugs in melanoma^90^.

### Prediction of immunotherapy response and analysis of therapeutic benefit in different risk groups

**Figure 10:**
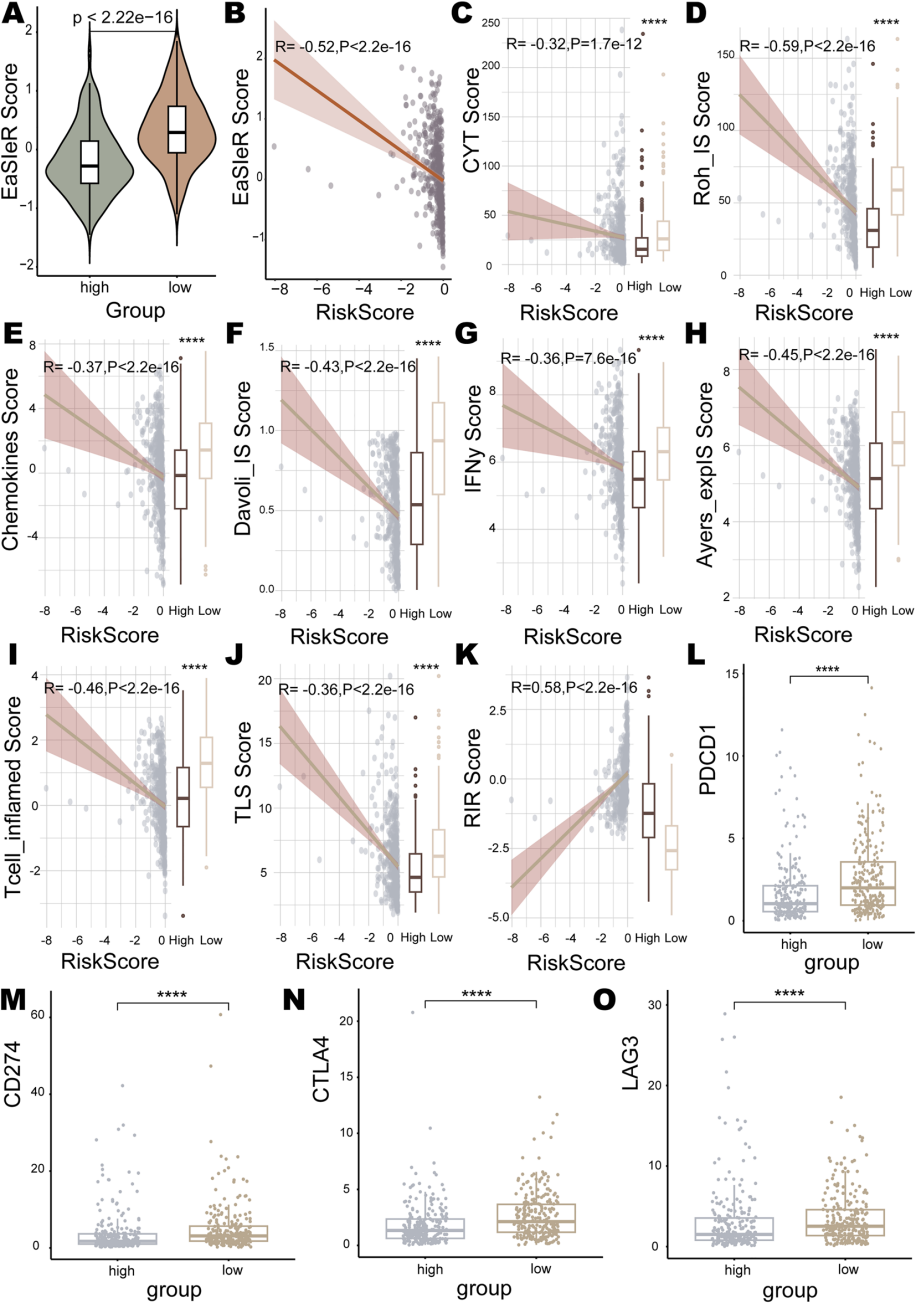
Immunotherapy response prediction for different risk groups. (**A**) Immune checkpoint inhibitors (ICI) treatment response scores for high and low risk groups. (**B**) Correlation of the patients' risk score with the ICI treatment response score. Correlation of the risk score with the scores of (**C**) CYT, (**D**) Roh_IS, (**E**) chemokines, (**F**) Davoli_IS, (**G**) IFNy, (**H**) Ayers_expIS, (**I**) Tcell_inflamed, (**J**) TL, and (**K**) RIR. Expression levels of immune checkpoints (**L**) PD-1, (**M**) PD-L1, (**N**) CTLA4, and (**O**) LAG3 in high-risk and low-risk groups.

The immune checkpoint blockade therapy has made revolutionary progress in the treatment of human cancers, including LUAD^91^. Hence, we employed the “EaSIeR” to predict the response scores to ICI therapy in the high-risk and low-risk groups. It was shown that the ICI treatment response score of the low-risk group was significantly higher than that of the high-risk group (*p* < 2e−16) (Fig. 10A). And the score was significantly negatively correlated with the risk score (R = − 0.52, *p* < 2.2e−16) (Fig. 10B). Additionally, “EaSIeR” further provided nine indicators (CYT, Roh_IS, chemokines, Davoli_IS, IFNy, Ayers_expIS, Tcell_inflamed, TLS, and RIR) for the evaluation of the immune response. Eight of these scores, except the RIR score, were higher in the low-risk group compared to the high-risk group, and these eight scores were negatively correlated with the risk score (Fig. 10C–K). This indicated a stronger immune response in the low-risk group. For the common immune checkpoints PD-1, PD-L1, CTLA4 and LAG3, inhibitors targeting them have already received regulatory approval or are undergoing clinical trials^92^. The expression levels of *PD-1* (*p* = 3.1e−08), *PD-L1* (*p* = 9.7e−09), *CTLA4* (*p* = 1.4e−08), and *LAG3* (*p* = 8e−05) were higher in the low-risk group (Fig. 10L–O). These portend that patients in the low-risk group may benefit more from immune checkpoint blockade.

**Figure 11:**
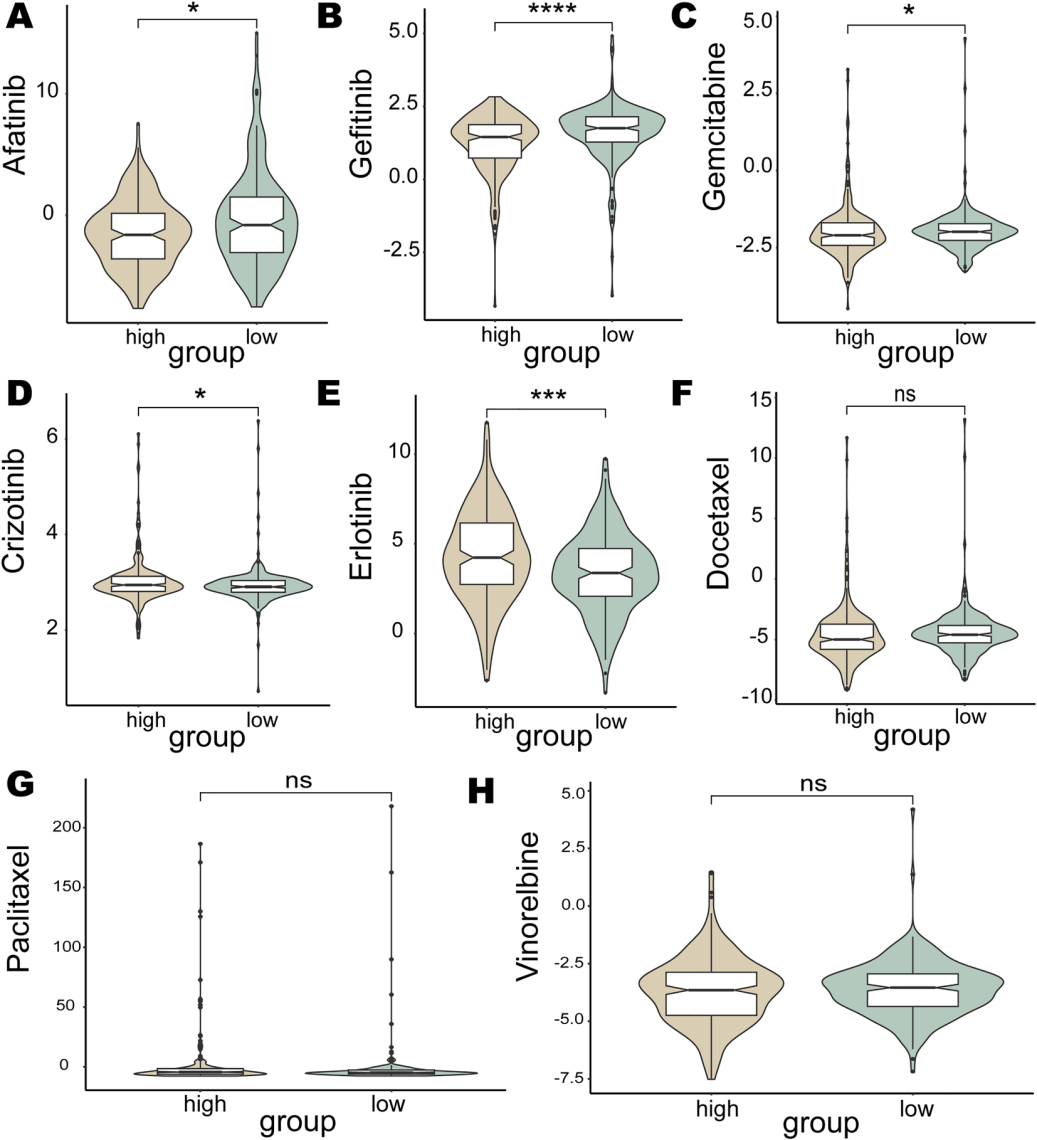
Benefit analysis of known therapeutic drugs in different risk groups. Sensitivity of high-risk and low-risk groups to known therapeutic drugs: (**A**) Afatinib, (**B**) Gefitinib, (**C**) Gemcitabine, (**D**) Crizotinib, (**E**) Erlotinib, (**F**) Docetaxel, (**G**) Paclitaxel, and (**H**) Vinorelbine.

The pRRophetic algorithm was applied to evaluate sensitivity differences between the high- and low-risk groups to drugs in the Cancer Genome Project database, in which eight NSCLC therapeutic drugs were included^93^. The results showed that the high-risk group had significantly lower IC50 values than the low-risk group for Afatinib (*p* = 1.9e−2), Gefitinib (*p* = 7.5e−06), and Gemcitabine (*p* = 5e−2), suggesting that high-risk patients may benefit more from these drugs (Fig. 11A–C). Crizotinib (*p* = 2.8e−2) and Erlotinib (*p* = 2e−04) had lower IC50 values in the low-risk group, indicating that these drugs may be more suitable for low-risk patients (Fig. 11D–E). However, there was no significant difference in sensitivity to Docetaxel, Paclitaxel, and Vinorelbine between the two groups (Fig. 11F–H).

## Discussion

LUAD is a cancer with a poor prognosis^94^, and the search for available LUAD biomarkers is important to achieve effective medical management of patients and improve their prognosis. In this study, we proposed an interpretable deep learning framework based on VAE, MLP, and SHAP that has mapped a large number of genes to a low-dimensional latent representation, enabling efficient feature selection. Considering the SHAP value reflected the influence of the feature genes as well as the positivity and negativity of the influence, a potential biomarker scoring algorithm was developed and 19 LUAD biomarkers were successfully identified. These biomarkers were enriched in the metabolic and immune pathways, revealing that LUAD patients present significant immune alterations and metabolic disturbances.

Given the importance of prognostic prediction in guiding precise therapeutic decisions, a 3-gene prognostic risk model for LUAD was developed through functional profiling and survival significance testing of identified biomarkers. The high- and low-risk groups divided by the risk score showed differences in the immune and metabolic landscapes. The low-risk group featured higher immune pathway activity, immune infiltration, and ICI treatment response scores. This indicates that high-risk patients were under an immunosuppressive state and that low-risk patients may be more suitable for ICI therapy. Interestingly, the three prognostic risk factors were positively related to the majority of immune-related genes. They were differentially expressed genes between the high-risk and low-risk groups, enriched in immune-related pathways such as regulation of T cell activation and leukocyte cell-cell adhesion, especially among them HLA-DRB1 and HLA-DRB5, which are immune checkpoints. Anti- PD-1/PD-L1 has yielded significant benefits for patients with NSCLC by inhibiting immune checkpoint activity, and the study demonstrated that HLA-DRB1 enhances the efficacy of anti-PD-1 therapy^90^. These suggest that the screened prognostic risk factors may be promising targets for immunotherapy of LUAD. Whereas the high-risk group presented significant enrichment of metabolic pathways. The high-risk patients had a higher propensity for metabolic reprogramming of aerobic glycolysis, glutamine, methionine, serine, and glycine, while having a lower propensity for metabolic reprogramming of arginine, tryptophan, fatty acids, and sphingolipids. Aerobic glycolysis and the metabolism of serine and glycine were related to poorer prognosis in cancers^71,80^. The close relationship between the prognostic risk model and metabolic reprogramming implies that targeting metabolism may contribute to improving the clinical prognosis of LUAD patients. Notably, increasing evidence suggests that altered metabolic patterns not only provide a survival advantage for tumor cells, but are also critical in modulating anti-tumor immune responses^95,96^ Methionine depletion and metabolic byproducts of aerobic glycolysis led to antitumor immune impairment^79,97^. Blocking glutamine metabolism has been shown to enhance anticancer immunity^98^. The study by Omkar et al.^99^ revealed that modification of fatty acid metabolism in CAR-T cells can improve their anti-tumor immune capacity. These findings revealed that the immune system and metabolism influence each other in cancer progression.

Angiogenesis, EMT, tumorigenic cytokines, and inflammation are correlates of tumor development. For the risk groups divided by risk score, the high-risk group presented higher angiogenic activity and lower EMT, tumorigenic cytokines, and inflammatory response. Studies have shown that angiogenesis is a poor prognostic indicator for survival in NSCLC^100,101^. The significant variations of these factors between the high- and low-risk groups may provide ideas for the treatment of LUAD. Of concern, EMT can stimulate cancer cells to produce proinflammatory factors, while inflammation can in turn induce EMT^102^. Not only that, EMT is also closely related to anti-tumor immune response^103^. In addition, researchers have found that the tumor vessel and the immune system affect each other's function, and that the combination of anti-angiogenesis therapies and immunotherapies may produce better therapeutic outcomes^104^. Aerobic glycolysis is associated with angiogenesis, a phenomenon closely associated with tumors^105^. Researchers have found that production of the tumorigenic cytokine IL-5 is associated with an enhanced response to immune checkpoint blockade in breast cancer patients^106^. Processes such as immune, metabolism, EMT, inflammation, and angiogenesis may crosstalk with each other, and combinatory interventions on them may present promising strategies for potential synergy.

To further explore the diagnostic potential of identified biomarkers, five common classification models were used, including a modified version of the integrated model AdaBoost.M1^39^, the generalized linear model GLM^107^, the L2 regularized linear support vector machine with class weights SVMLinearWeights2^108^, the penalized logistic regression model PLR^109^ and the extreme gradient boosting model XGBDART^110^. The five classification models were trained and evaluated on the TCGA dataset and another independent dataset GSE81089 from GEO (involving 108 LUAD samples and 19 normal samples) in a leave-one-out method. The models were trained using the R package “caret” and the AUC was used as the evaluation metric for the models. For the TCGA LUAD dataset, the AUC values of the AdaBoost.M1, GLM, SVMLinearWeights2, PLR, and XGBDART models were 0.9012, 0.875, 0.8929, 0.891, and 0.8918, respectively (Supplementary Fig. S3A). For the GSE81089 dataset, the AUC values for the five classification models reached 0.8855, 0.9769, 0.9242, 0.9454, and 0.8684, respectively (Supplementary Fig. S3B). To better understand the performance of the models, performance metrics including accuracy, precision, recall, and F1-score were also calculated. The five classification models achieved accuracy, precision, recall, and F1-score of over 94% on both the TCGA dataset and the GSE81089 dataset (Supplementary Table S1). The excellent performance of multiple classification models on different datasets reflected the diagnostic superiority of the biomarkers for LUAD.

## Conclusion

Collectively, this study proposed a novel deep learning framework and successfully identified 19 biomarkers and 3 prognostic signatures of LUAD. The immune and metabolic landscapes as well as alterations in angiogenesis, EMT, tumorigenic cytokines, and inflammation based on prognostic signatures were investigated. In the meantime, the prognostic signatures may be indicators for predicting response to immunotherapy, but further research is needed to clarify this point. Our study may provide new insights for screening genomic markers for LUAD, provide support for clinical judgment of LUAD prognosis and have potential to be applied to other cancers.

### Supplementary Information

Supplementary Information.

## Data Availability

All data generated or analysed during this study are included in this published article (and its Supplementary Information files).
