# Supplementary material for: Immune, metabolic landscapes of prognostic signatures for lung adenocarcinoma based on a novel deep learning framework
Source: Sci Rep. 2024 Jan 4;14:527. doi: 10.1038/s41598-023-51108-x (PMC10767103; doi:10.1038/s41598-023-51108-x)
Supplement: Supplementary file 1 — Supplementary Information. [file 41598_2023_51108_MOESM1_ESM.pdf]

Immune, metabolic landscapes of prognostic signatures for lung adenocarcinoma based on a novel deep learning framework

Shimei Qin [1,+], Shibin Sun [1,+], Chengcheng Yang [2,+], Yahui Wang [1], Chao Li [1], Junjie Lv [1], Lei Fu [1], Ming Wu [1], Jinxing Yan [1], Xinyan Wang [2,*], Lina Chen [1,*]

Supplementary Materials

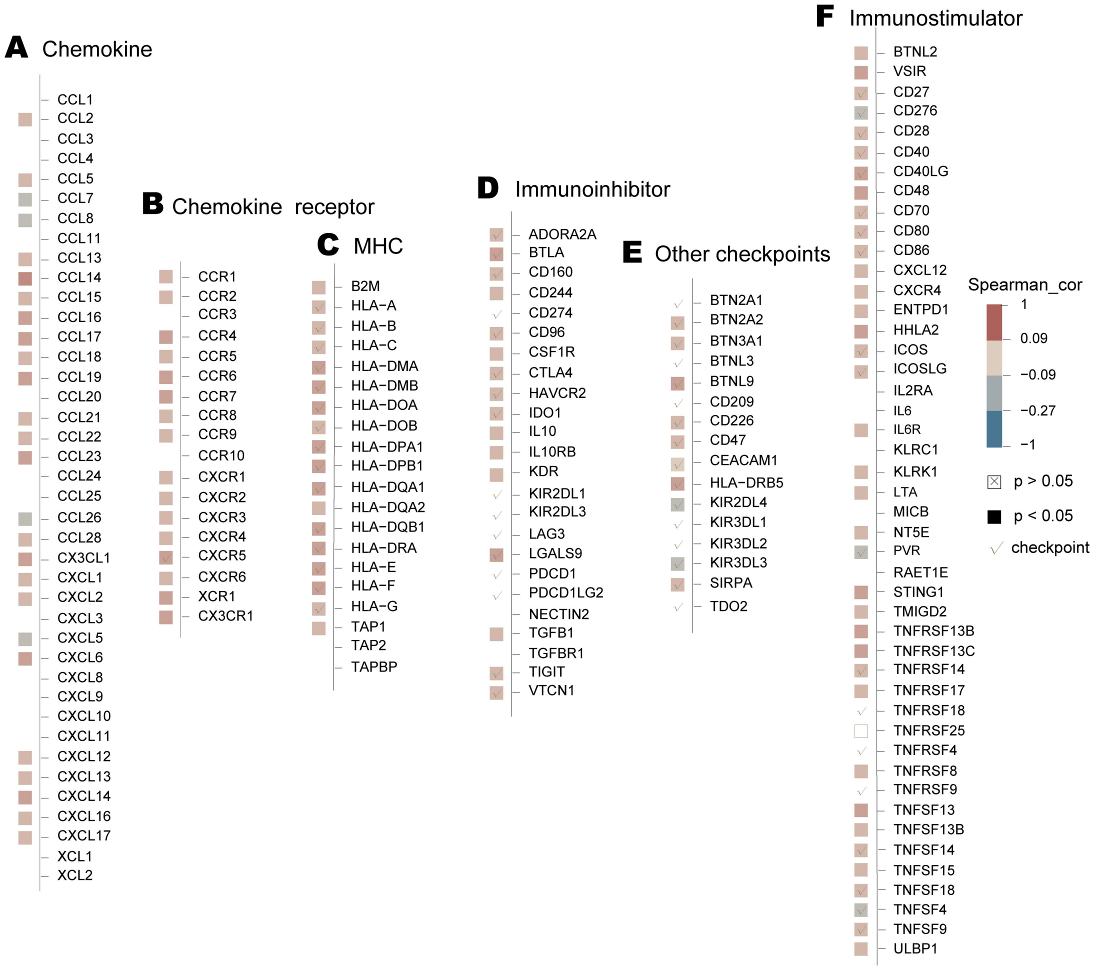

Figure S1

The correlation of *SCGB1A1* with immune-related genes and immune checkpoints. **(A)** Chemokine genes. **(B)** chemokine receptor genes. **(C)** MHC. **(D)** immunosuppressive genes. **(E)** other immune checkpoints. **(F)** immunostimulatory genes.

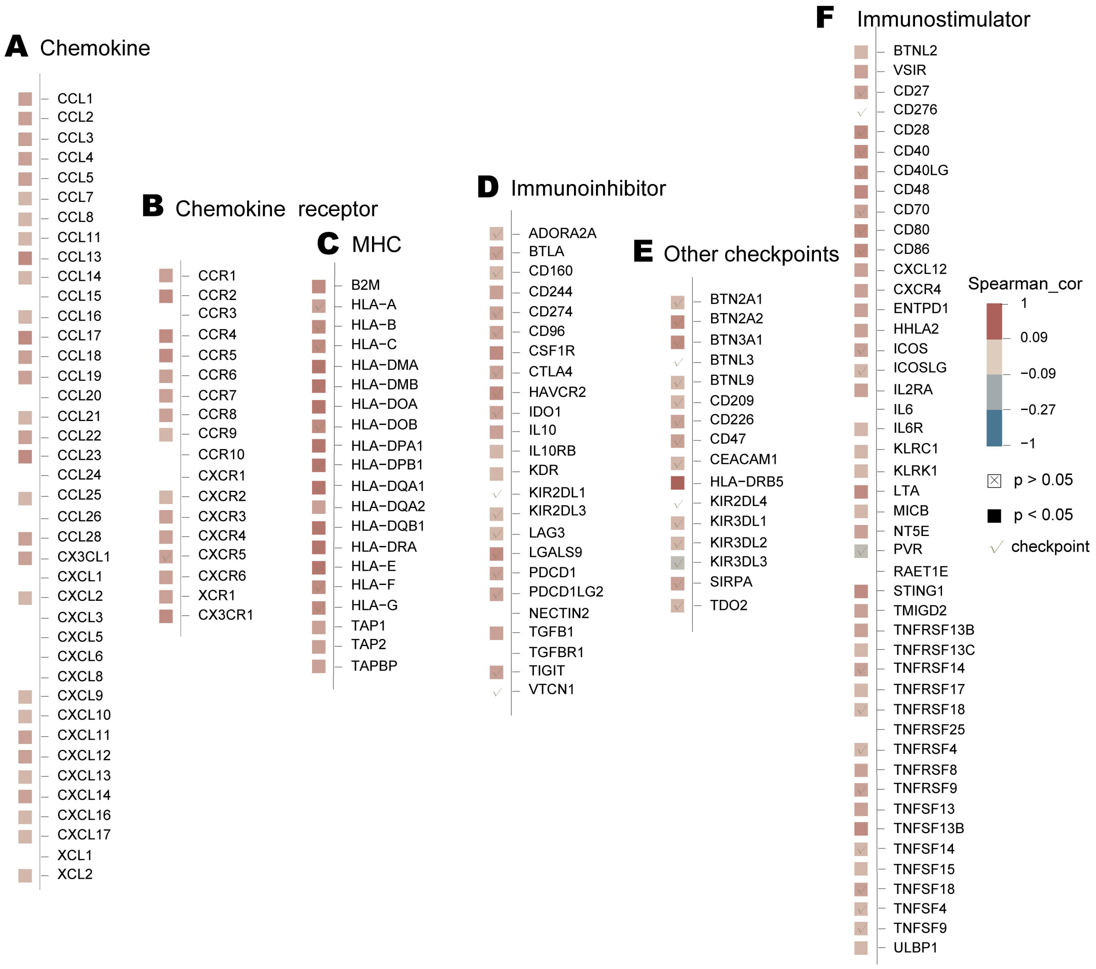

Figure S2

The correlation of *HLA-DRB5* with immune-related genes and immune checkpoints. **(A)** Chemokine genes. **(B)** chemokine receptor genes. **(C)** MHC. **(D)** immunosuppressive genes. **(E)** other immune checkpoints. **(F)** immunostimulatory genes.

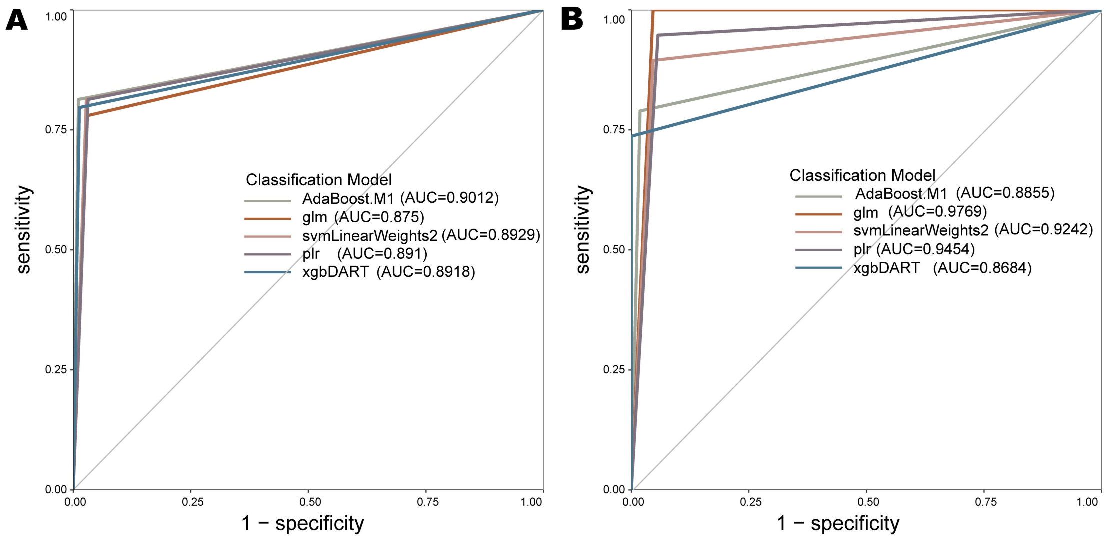

Figure S3

The receiver operating characteristic curves of the five classification models applied to different datasets. **(A)** The TCGA dataset. **(B)** The GSE81089 dataset.

Table S1

Performance metrics of the five classification models.

Model	Dataset	Accuracy	Precision	Recall	F1-score
AdaBoost.M1	TCGA	0.9716	0.9798	0.9889	0.9843
AdaBoost.M1	GSE81089	0.9528	0.9636	0.9815	0.9725
glm	TCGA	0.9515	0.9757	0.9703	0.973
glm	GSE81089	0.9606	1	0.9537	0.9763
svmLinearWeights2	TCGA	0.9565	0.9794	0.9722	0.9758
svmLinearWeights2	GSE81089	0.9449	0.981	0.9537	0.9671
plr	TCGA	0.9532	0.9794	0.9685	0.9739
plr	GSE81089	0.944	0.9901	0.9434	0.9662
xgbDART	TCGA	0.9682	0.9779	0.987	0.9825
xgbDART	GSE81089	0.9606	0.9558	1	0.9774
